# Impact of Co-Culture on the Metabolism of Marine Microorganisms

**DOI:** 10.3390/md20020153

**Published:** 2022-02-21

**Authors:** Flore Caudal, Nathalie Tapissier-Bontemps, Ru Angelie Edrada-Ebel

**Affiliations:** 1 Laboratoire Biotechnologie et Chimie Marines, Université Bretagne Sud, EA3884, LBCM, IUEM, CEDEX, 56321 Lorient, France; flore.caudal@univ-ubs.fr; 2 CRIOBE, USR3278-EPHE/CNRS/UPVD/PSL, University of Perpignan via Domitia, 52 Avenue Paul Alduy, 66860 Perpignan, France; nathalie.tapissier@univ-perp.fr; 3 Laboratoire d’Excellence ‘CORAIL’, Moorea 98729, French Polynesia; 4 The Natural Products Metabolomics Group, Strathclyde Institute of Pharmacy and Biomedical Sciences, Faculty of Science, University of Strathclyde, The John Arbuthnott Building, 161 Cathedral Street, Glasgow G4 0RE, UK

**Keywords:** co-culture, natural products, marine bacteria, marine fungi

## Abstract

Natural products from plants have been listed for hundreds of years as a source of biologically active molecules. In recent years, the marine environment has demonstrated its ability to provide new structural entities. More than 70% of our planet’s surface is covered by oceans, and with the technical advances in diving and remotely operated vehicles, it is becoming easier to collect samples. Although the risk of rediscovery is significant, the discovery of silent gene clusters and innovative analytical techniques has renewed interest in natural product research. Different strategies have been proposed to activate these silent genes, including co-culture, or mixed fermentation, a cultivation-based approach. This review highlights the potential of co-culture of marine microorganisms to induce the production of new metabolites as well as to increase the yields of respective target metabolites with pharmacological potential, and moreover to indirectly improve the biological activity of a crude extract.

## 1. Introduction

Secondary metabolites are molecules found in nature that are produced by organisms from one or several primary metabolites. Secondary metabolites are not required for basic viability but are essential for the organism’s survival strategies. These natural products are synthesised by many organisms, such as bacteria, fungi, plants, and sponges, and are found in all environments, both terrestrial and marine.

Natural products from terrestrial plants and microorganisms are a common source of modern drug molecules. Historically, they have been used since ancient times and in traditional medicine for the treatment of many diseases and illnesses. Natural products constitute the foundation of medicines for human health. The earliest records dating from around 2600 BC documented the use of about 1000 substances derived from plants in Mesopotamia, such as oils of *Cupressus sempevirens* (cypress) and *Glycyrrhiza glabra* (liquorice). One of the best-known documents is the “*Ebers Papyrus*”, dating from 1500 BC, recording more than 700 medicines, most of them of plant origin [1,2,3].

Recently, Newmann and Cragg (2012) reported that between 1981 and 2010, 48.6% of the 175 small drug molecules used in cancer therapy were either natural products or derived therefrom. In other areas, such as anti-infectives, the influence of natural products as lead structures has been quite remarkable [4,5].

Regarding microorganisms, it is estimated that the number of existing microbial species would be around 10^5^ to 10^6^, but only several thousand have been isolated into pure cultures [6]. It is still possible to find biologically active molecules with novel structures or unprecedented carbon skeletons and unique modes of action from uncultivable and undiscovered species. Additionally, unlike macro-organisms, microorganisms have significant advantages in terms of their resilience and feasibility in terms of the cost of an industrial-scale fermentation. For the preservation of their environment of origin, the possibility to collect samples sustainably without having an impact on the biodiversity of the natural environment is of great importance. Furthermore, microorganisms are a prolific source of structurally diverse bioactive metabolites and have yielded some of the most important antibiotics in the pharmaceutical industry—one best known example is the discovery of penicillin by Fleming in 1928 [2].

Plants have also been proven to be very good sources of bioactive natural products, but in recent years, the marine environment has demonstrated its ability to provide new structural entities as well. More than 70% of our planet’s surface is covered by oceans, and in certain marine ecosystems, such as coral reefs, experts estimate that the biological diversity is higher than in a tropical rainforest [7]. Exploration of the marine environment has for a very long time been limited to coasts and ocean surfaces, but with technical advances in diving and remotely operated vehicles (ROVs), it is becoming easier to take samples at deeper ocean depths. As for terrestrial microorganisms, marine bacteria and fungi quickly proved to be prolific sources of novel bioactive compounds [8].

**Figure 1:**
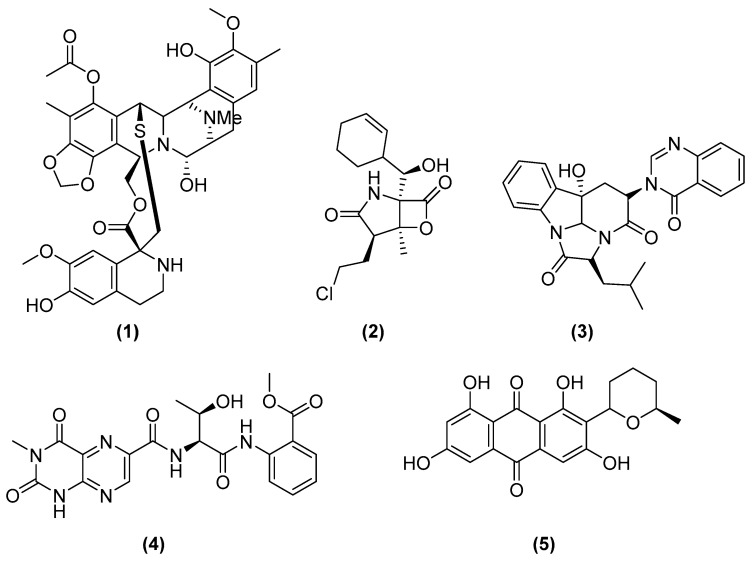
Chemical structures of trabectedin (**1**), salinosporamide A (**2**), isopropylchaetominine (**3**), isoterrelumamide A (**4**), and 5′-epi-averufanin (**5**).

Therapeutic molecules from the marine environment are now available in the market. In 2007, trabectedin (**1**) (Yondelis^®^) was the first marine-derived anti-neoplastic drug to be approved in the European Union. Trabectedin (Figure 1) was initially isolated from the Caribbean tunicate *Ecteinascida turbinata* [9]. Currently, PharmaMar commercially prepares Yondelis^®^ by chemical synthesis [10,11]. Another example is salinosporamide A (**2**) (Marizomib^®^), which was isolated from a marine-derived microorganism. In 2002, Feling et al. of the Fenical and Jensen group initially isolated salinosporamide A (**2**) (Figure 1) from a sediment obligate marine actinomycete, *Salinospora tropica* [12]. Salinosporamide A (**2**) is a 20S proteasome inhibitor that was granted an orphan drug designation by the European Medicines Agency (EMA) for the treatment of multiple myeloma [13]. 

Natural products therefore represent a non-negligible share of molecules on the market, but currently, the risk of rediscovery is always high. They have been laid aside for several years by the pharmaceutical industry in favour of synthetic molecules. However, with the discovery of silent gene clusters and the significant improvement of screening, analytical, and molecular biological techniques, a renewed interest in natural products research has been increasingly flourishing. 

Whole-genome sequencing of different microorganisms has disclosed inconsistencies between the number of groups of genes identified using informatic approaches and the number of metabolites produced by the organisms [14]. The genes that are present but not expressed under laboratory conditions are called silent gene clusters. A hypothesis was then proposed for activating these silent gene clusters to potentially access new secondary metabolites that could be of pharmacological interest. Different strategies for activating these silent gene clusters are described below.

Various strategies have been put together to influence the production of secondary metabolites by microorganisms when grown in the laboratory. As described in a 2014 review paper by Bertrand et al., it is possible to explore organisms at different levels, from their genome to their metabolome via their transcriptome and proteome [15]. At the genome level, metabolic engineering was among the first techniques to be exploited, and was defined by Khosla and Keasling as “the process of rerouting metabolic pathways by genetic manipulation” [16]. In recent years, metabolic engineering has enabled the development of several techniques of genetic manipulation, the most common of which involves the biosynthesis of secondary metabolites in a heterologous host. When the natural producer of certain targeted secondary metabolites do not grow well under laboratory or industrial conditions, the biosynthetic gene cluster of interest is inserted into a different but more robust microorganism, which is selected on the basis of the involved biosynthetic pathway of targeted metabolites [17]. Mutasynthesis is another technique in which a mutant microorganism that is deficient in a key part of the biosynthetic pathway is employed and to which analogous precursors are supplemented to augment the production of new metabolites of interest [18]. These methodologies applied on the microbial genome have also been utilised along with combinatorial biosynthesis. With combinatorial biosynthesis, genes and modules of closely related synthetic pathways of secondary metabolites would be rearranged to create new congeners [17]. The success rate of combinatorial synthesis depends on the position of the introduction of the modification. Modifications at the later stages of the synthetic pathway would afford higher success rates. Indeed, there will potentially be less enzyme downstream of the point of alteration, due to having to tolerate these modifications of the substrate and complicating the process [17]. For this kind of method, it is essential that the biosynthetic gene clusters of the microorganisms are sequenced and the functions of the genes of interest are also assigned [15].

Epigenetic modification is another commonly used strategy utilised on the transcriptome and the proteome, where microorganisms were treated with epigenetic modifiers such as DNA methyl transferase or histone deacetylase inhibitors to modulate the DNA histone remodelling the chromatin for initiating the transcription of silent genes [19]. 5-Azacytidine is among the most commonly used DNA methyl transferase inhibiting molecules. Akone et al. (2016) of the Proksch group in Düsseldorf, Germany, used 5-azacytidine on fungal endophytic cultures of *Chaetomium* sp. to generate five additional polyketide congeners along with enhancing the production of targeted anticancer metabolites produced in their co-culture experiments [20]. Alternatively, the Oshima group utilised suberoyl bis-hydroxamic acid, an example of a histone deacetylase inhibitor that afforded the production of a novel series of three new prenylated tryptophan analogues known as luteorides from cultures of the entomopathogenic fungus, *Torrubiella luteorostrata* [21]. Epigenetic modification has demonstrated the accumulation of new natural products of interest. 

Microbial cultures grown under laboratory conditions may afford a different metabolomic profile when found in their original environment, and therefore it is important to take into account the chemical–ecological relationships that occur in their communities [15]. Moreover, genomic data are not available for all microorganisms. Therefore, non-genome-dependent techniques have also been developed. One of the strategies often related to chemical ecology is a cultivation-based approach, namely, the “one strain many compounds” (OSMAC) technique, which underlines how a single strain can produce different molecules when grown under different inoculation and incubation conditions [14]. Different parameters can be changed, such as the salinity of the medium, its composition, the addition of different salts, elicitors, differences in temperature or agitation conditions, and many more. One of the other parameters to be taken into account is also the observed differences in metabolomic profiles between cultures grown in solid and liquid media, as illustrated in a review paper by Romano et al. (2018) of the Dobson group in Cork, Ireland [14]. An example to illustrate the OSMAC approach is a comparative study on the changes in the metabolomic profile of a sponge-associated fungus, *Aspergillus carneus* [22]. In this study, Özkaya et al. (2018) of the Proksch group isolated three new natural products (Figure 1)—isopropylchaetominine (**3**), isoterrelumamide A (**4**), and 5′-epi-averufanin (**5**)—after the fungus was inoculated on three different media. Isoterrelumamide A (**4**) was present only after fermentation on modified Czapek medium, while 5′-epi-averufanin was only afforded when incubated on solid rice medium with or without sea salt. It can thus be seen that depending on the composition of the culture medium in which the microorganism was inoculated, the detected secondary metabolites were not always the same [22].

Finally, the third option is by co-culture, also called mixed-fermentation, which is another cultivation-based approach. In this technique, two or more different microorganisms are inoculated together to mimic the natural habitat in which symbiotic or competing interactions between microorganisms are simulated and/or enhanced. Natural habitats could be characterised by limited access to resources and nutrients, as well as by an exchange of metabolites between micro- and/or macroscopic organisms. Competition between microbes is deliberately provoked by inducing stress factors, which stimulates the activation of silent gene clusters that were not expressed under classical culture conditions [8].

In this review, we focus on secondary metabolites from marine microorganisms and how co-culture techniques can induce their production. The different techniques implemented for mixed fermentations are discussed. Finally, the review is concluded through a critical enquiry on the potential for producing new metabolites by employing different novel co-culture techniques. This review, however, will not differentiate marine fungi from marine-derived fungi. Marine-derived fungi are species that do not belong to the well-documented lineages of obligate marine fungi, or when the source macro-organism cannot be conclusively identified as marine [23]. Nevertheless, these microorganisms could be isolated from oceans; ocean-dwelling animals; marine algae; and from marine–terrestrial transitional habitats, e.g., mangroves and other halophytes.

## 2. Co-Cultivation between Marine Microorganisms

The different results obtained from various representative examples of co-culture conditions presented in publications from 2010 to 2020 were surveyed in this review paper. The next three sections will tackle, and present publications found under the search terms: marine culture “coculture” OR “mixed fermentation” OR “co-culture” AND “fungi” OR “bacteria”. Articles were then classified into the three sections according to the type of co-inoculated microorganisms: (1) bacteria–fungi, (2) between bacteria, and (3) between fungi.

### 2.1. Co-Cultures between Fungi and Bacteria

In this section, only the three most relevant sample articles were found to correspond to the mixed fermentation of marine fungi and bacteria. One example of the association between marine-derived fungi and a terrestrial bacterium is also presented.

**Figure 2:**
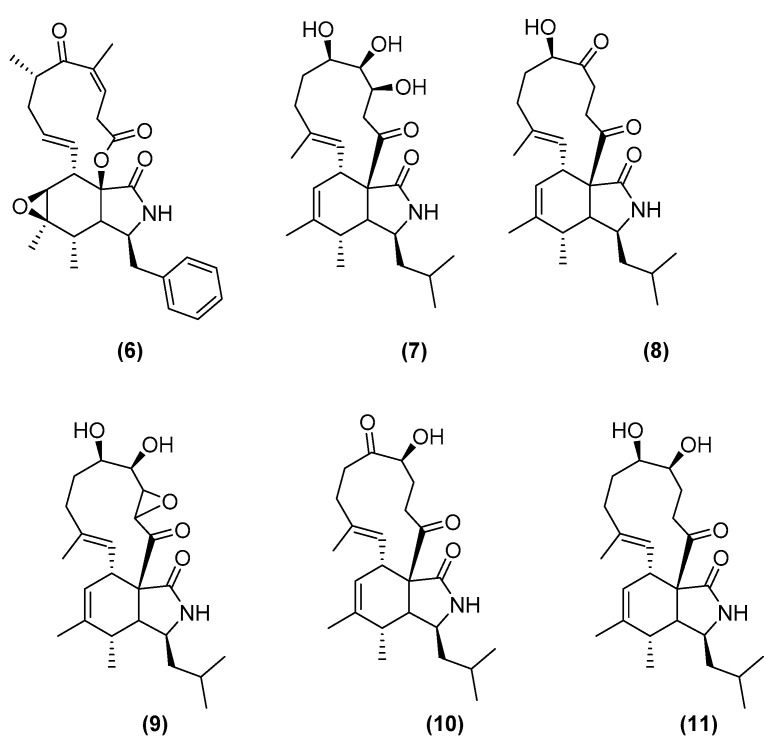
Chemical structures of rosellichalasin (**6**), aspochalasin E (**7**), aspochalasin P (**8**), aspochalasin H (**9**), aspochalasin M (**10**), and 19,20-dihydro-aspochalasin D (**11**).

Yu, Ding, and Ma (2016) co-cultured the fungus *Aspergillus flavipes* with the actinomycete *Streptomyces* sp., which were both isolated from marine coastal sediment from the Nanji Islands in China. The mixed fermentation increased the production yield of new cytochalasin analogues from the corresponding fungal monoculture [24]. This co-culture induced the production of six cytochalasans shown in Figure 2, namely, rosellichalasin (**6**), aspochalasin E (**7**), aspochalasin P (**8**), aspochalasin H (**9**), aspochalasin M (**10**), and 19,20-dihydro-aspochalasin D (**11**). These secondary metabolites were produced in monocultures of *Aspergillus flavipes* but at very low concentrations that were below the limit of quantification through HPLC-TOF-MS. Yu, Ding, and Ma (2016) detected the respective *m*/*z* ion peaks, demonstrating the enhancement of cytochalasan production that required physical contact between non-inactivated co-cultivated microbes. All six cytochalasans exhibited strong activity against *Streptomyces* sp. with an inhibition rate of 50–80% at concentrations between 2 and 16 μg/mL but had no effect on the fungus *A. flavipes* at the same concentration. This result indicated that the cytochalasans assisted *A. flavipes* to compete with *Streptomyces*.

**Figure 3:**
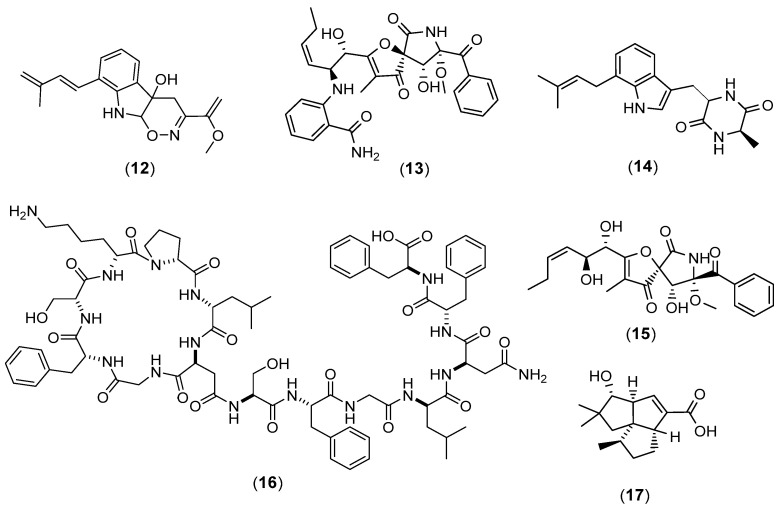
Chemical structures of luteoride D (**12**), pseurotin G (**13**), terezine D (**14**), 11-*O*-methylpseurotin A (**15**), chaxapeptin (**16**), and pentalenic acid (**17**).

From the same perspective, Wakefield et al. (2017) of the Jasper group in Aberdeen, United Kingdom, described co-cultures of a marine-derived fungus, *Aspergillus fumigatus* MR2012, with two hyper-arid desert bacterial isolates *Streptomyces leeuwenhoekii* strain C34 and strain C58 [25]. The co-culture with strain C34 afforded a new luteoride derivative, luteoride D (**12**); a new pseurotin congener, pseurotin G (**13**); and two known compounds, terezine D (**14**) and 11-*O*-methylpseurotin A (**15**), which were not found in the axenic fungal culture (Figure 3). Interestingly, some of the major fungal metabolites produced by the respective axenic cultures were suppressed. The microbial co-culture with strain C58 dramatically increased the production of chaxapeptin (**16**) that was already initially found in the axenic culture of the bacteria while inducing the production of a known pentalenic acid (**17**). It is interesting to note that the co-culture process could induce the production of new compounds and increase the yield of existing metabolites from both axenic microbial cultures and not in only one of the two microorganisms involved in the co-culture. However, co-culturing could also result in a loss of production of some major families of fungal metabolites present in its axenic culture, while none of the bacterial metabolites were proven to have antifungal effects.

To observe the induction of antimicrobial compounds as well as the production of biosurfactants and quorum-sensing inhibitors, Dusane et al. (2011), from Pune University in India, worked with four marine epibiotic bacteria (*Bacillus* sp. S3, *Bacillus pumilus* S8, *Bacillus licheniformis* D1, and *Serratia marcescens* V1) that exhibited bioactivity against pathogenic or biofouling fungi [26]. Dusane et al. demonstrated the induction and/or enhancement of antimicrobial activity by co-culturing marine epibiotic bacteria with *Candida albicans* and *Yarrowia lipolytica*. Except for S8, monocultures of S3, D1, and V1 displayed antifungal activity against *C. albicans*. On the other hand, none of the axenic cultures of epibiotic bacteria showed any antifungal activity against *Y. lipolytica*. However, cell-free supernatants of mixed fermentation cultures of marine *Bacillus* sp. S3 and *C. albicans* induced antifungal activity against *Y. lipolytica*, while co-cultures of D1 and *C. albicans* enhanced biosurfactant production. Dusane et al. illustrated the induction and enhancement of the production of certain metabolites of interest with specific biological activity by co-culturing microorganisms with pathogenic microbial strains.

### 2.2. Co-Cultures between Two Bacterial Strains

In recent years, several reports on co-cultures between bacteria have been published. Based on the search terms mentioned above, we chose the 11 most appropriate publications reported on co-cultures between marine bacteria for further evaluation, including one of the papers presented at the end of the previous section. Bacteria in the marine environment are found in numerous habitats, not only in sediments, but also in association with algae and other macro-organisms. These microbes can have a free mode of life in water or sessile in the form of a biofilm in which bacteria can communicate by quorum sensing [27].

In the last paper presented in the previous section, Dusane et al. also co-cultured four marine epibiotic bacteria with other bacteria, such as *Pseudomonas aeruginosa* PA and *Bacillus pumilus* BP [26]. The respective cell-free supernatants of co-cultures of marine *Bacillus* sp. S3 and S8 with *Ps. aeruginosa* and *B. pumilus* allowed for the induction of antifungal activity. Again, antifungal activity was evident when marine isolates were co-cultivated with either pathogenic or biofouling bacterial cultures. However, the enhancement of the antibacterial activity was less apparent when compared with the distinctive induction of antifungal activity by the cell-free supernatants of the co-cultures. Exceptionally, the antibacterial activity of *B. licheniformis* D1 against *Ps. aeruginosa* was significantly increased when co-cultured with the latter pathogen. Similarly, a significant enhancement of biosurfactant production was observed when *S. marcescens* V1 was co-cultivated with the biofouling bacteria *B. pumilus*, which was not observed when inoculated with other non-biofouling marine bacterial isolates. On the other hand, cell-free supernatants of co-cultures of *Ps. aeruginosa* and *Bacillus* sp., as well as of their respective axenic cultures, were also observed to exhibit quorum sensing inhibitory activity. Co-cultivation of marine epibiotic bacteria with pathogenic and fouling bacteria were able to either induce or enhance all the indicated bioactivity in this research paper, which signposted the production of secondary metabolites with antibiotics, anti-quorum sensing, and biosurfactant activities. 

In another study, Haque et al. (2016) focused on the antifungal and anticancer activity of an ethyl acetate crude extract obtained from a co-culture of two marine *Streptomyces* sp., ANAM-5 and AIAH-10, isolated from mangrove forest soil samples of Sundarbans, Bangladesh [28]. Only crude extracts of the co-cultures were implemented for the bioassays, while no isolated purified compounds could be utilised. The crude extracts exhibited antifungal activities against both *Saccharomyces cerevisiae* and *Aspergillus niger* with an MIC value of 64 µg/mL as well as against *Candida albicans* with an MIC value of 32 µg/mL. Haque et al. evaluated the antineoplastic activity of the co-culture extracts by measuring cell growth inhibition and the enhancement of the life span of Ehrlich ascites carcinoma (EAC) cell-bearing mice. The efficiency of the activity of the crude extract was compared with the standard anticancer drug bleomycin. Co-cultured bacterial crude extracts effectively inhibited cell growth rate at 75.75% at a of dose 100 mg/kg (i.p.), which is quite comparable to that of bleomycin at 0.3 mg/kg. Crude extracts of the co-cultured bacteria also effectively increased the life span of tumour-bearing mice by 71.79% at a dose of 100 mg/kg (i.p.). In conclusion, the extract obtained from the co-culture displayed interesting antimicrobial and anti-neoplastic activity. Reported biological activity was enhanced by 70% with the crude extracts of the co-culture, but there is no record whether new compounds were generated or merely enhanced concentration of biologically active components. 

Ravi et al. (2017), from VIT University in India, briefly described co-culturing marine and terrestrial actinomycetes to enhance anticancer bioactivity [29]. The co-cultured bacterial extract gave an IC_50_ value of 20 µg/mL, while the extract from the monoculture afforded a weaker IC_50_ of 40 µg/mL. It was interesting to perceive that the co-culture of two actinomycetes species from different ecological origin could augment the production of potential anticancer metabolites. Ravi et al. also disclosed that under co-culture conditions, albeit with the occurrence of normal and healthy actinomycete filaments, spore production was greatly suppressed. 

**Figure 4:**
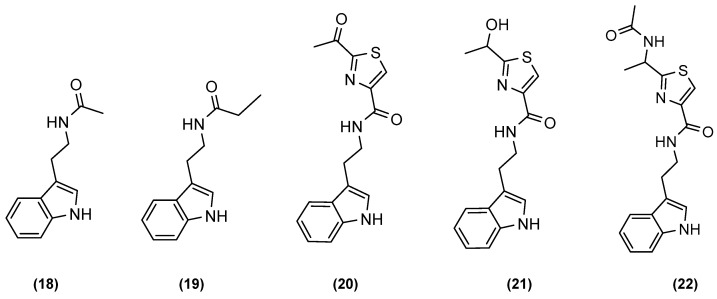
Chemical structures of the co-culture-induced compounds *N*-acetyltryptamine (**18**), *N*-propanoyltryptamine (**19**), bacillamide A (**20**), bacillamide B (**21**), and bacillamide C (**22**).

Yu et al. (2015) of the Ma group from Zhejiang University, Hangzhou, developed a relatively simple fermentation method to increase the production of algicidal tryptamine derivatives [30]. Tryptamine derivatives, as shown in Figure 4, were initially introduced as very promising molecules for the control of harmful algal blooms, but their application and further study was halted by the low production rate of the bioactive metabolites in axenic cultures of *Bacillus mycoides*. Bacterial co-cultures were established using the OSMAC technique to optimise the production of potent algicidal tryptamine congeners. Yu et al. subjected *Streptomyces* sp. CGMCC4.7185 and *Bacillus mycoides* to mixed fermentation, and various experiments were carried out to find the best culture medium and pH to enhance the production of the desired tryptamine analogues. The mean production yields of the respective tryptamine derivatives from a 68 L fermentation culture under optimised conditions were 14.9 mg/L for *N*-acetyltryptamine (**18**), 2.8 mg/L for *N*-propanoyltryptamine (**19**), 3.0 mg/L for bacillamide A(**20**), 13.7 mg/L for bacillamide B (**21**), and 9.6 mg/L for bacillamide C (**22**). The total tryptamine yield represented more than 50% of the crude ethyl acetate extract (3.0 g in the 5.5 g total extract). Under normal axenic culture conditions, the occurrence of various tryptamine analogues was undetectable by HPLC equipped with a diode array detector at a UV range between 200 and 400 nm but perceivable by TLC when visualised with iodine vapour. 

Low biomass yields with bacterial cultures are a frequent challenge. Under a co-culture environment, a higher production yield of the targeted secondary metabolites was facilitated. With higher yields, the various analogues can be further evaluated for their other potential activities and cytotoxicity studies. However, subsequently, for a large (industrial) scale-up, as the fermentation parameters can change and are not directly reproducible, then it may be necessary to employ other techniques or in combination with co-cultivation such as hemisynthesis or one of the metabolic engineering methods described above in Section 1. 

**Figure 5:**
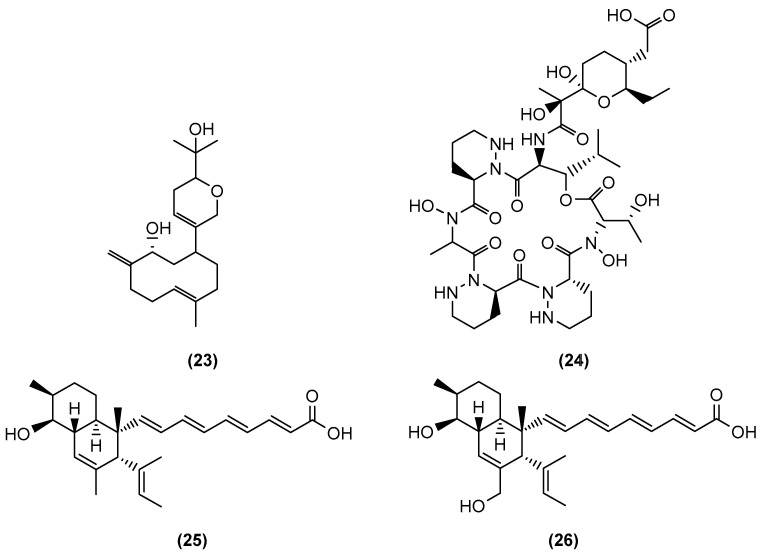
Chemical structures of lobocompactol (**23**), dentigerumycin E (**24**), and janthinopolyenemycins A (**25**) and B (**26**).

Cho and Kim (2012), from Soon Chun Hyang University, Korea, increased the production of a target antifouling compound by co-culturing a bacterium producing the metabolite of interest with a competitor bacterium isolated from the same host macroorganism [31]. The active antifouling diterpene lobocompactol (**23**) (Figure 5) was initially isolated from the marine *Streptomyces cinnabarinus* PK209. To increase lobocompactol production, Cho and Kim selected *Alteromonas* sp. KNS-16 as the lobocompactol-resistant bacterium for a co-culture competitor. Both bacterial strains, PK209 and KNS-16, were isolated from the surface of a seaweed rhizosphere. The co-culture of these two bacterial strains resulted in a 10.4-fold increase in the production of lobocompactol compared to the PK209 monoculture (2.7 mg/L instead of 0.25 mg/L). Lobocompactol (**23**) exhibited significant antifouling activity with an EC_50_ of 0.18 µg/mL against the macroalga *Ulva pertusa* and 0.43 µg/mL against the diatom *Navicula annexa*. The extract also demonstrated its activity against the fouling bacteria. These studies from Yu et al. [30] as well as Cho and Kim [31] presented the potential of co-cultures in increasing the production of a particular target metabolite.

Co-cultures have also led to the discovery of new bioactive molecules. Shin et al. (2018) from Seoul National University elucidated a new piperazic acid-bearing cyclic peptide, dentigerumycin E (**24**) (Figure 5), afforded by the co-culture of the marine *Streptomyces* sp. JB5 and *Bacillus* sp. GN1, isolated from an intertidal mudflat in Wando, Republic of Korea [32]. *Streptomyces* sp. JB5 was co-cultivated with seven different bacterial strains including *Bacillus* sp. HR1, *Paenibacillus* sp. CC2, *Brevibacillus* sp. PTH23, *Streptomyces* sp. SD53, *Streptomyces* sp. UTZ13, *Hafnia* sp. CF1, and *Mycobacterium* sp. Myc06. However, only the co-culture with *Bacillus* sp. HR1, which is phylogenetically close to *Bacillus* sp. GN1, afforded dentigerumycin. Although the mechanism triggering the biosynthesis of dentigerumycin E (**24**) by *Bacillus* strains remains unclear, the results of these co-culture experiments imply that *Bacillus* strains, which are most closely related to *B. cereus*, may share the ability to induce production of dentigerumycin E (**24**) in *Streptomyces* sp. JB5. Full genome sequencing of *Streptomyces* sp. JB5 allowed for the identification of the BGC for dentigerumycin E (**24**), and thus it was confirmed that it was indeed produced by the *Streptomyces* strain. Different experiments have shown that dentigerumycin E had antimetastatic potential against breast cancer cells. 

Anjum et al. (2018) of the Lian group from Zhejiang University, Hangzhou, isolated janthinopolyenemycins A (**25**) and B (**26**), two rare polyketides (Figure 5), from a co-culture of two *Janthinobacterium* spp. strains ZZ145 and ZZ148 obtained from a marine soil sample, and the strains were co-inoculated in different media including rice solid medium [33]. Both polyketides exhibited antifungal activity against *C. albicans* with MIC and MBC values of 15.6 and 31.25 µg/mL, respectively, although their bioactivities were less potent than the control (amphotericin B). The janthinopolyenemycin congeners were also found to be active against methicillin-resistant *Staphylococcus aureus* (MRSA) and *E. coli*. The co-culture of ZZ145 and ZZ148 induced the generation of the new janthinopolyenemycin congeners. 

**Figure 6:**
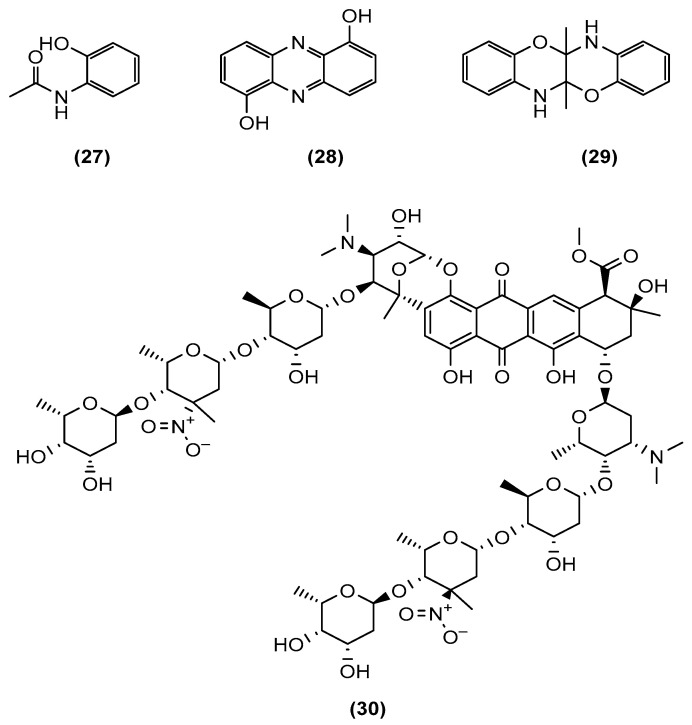
Chemical structures of *N*-(2-hydroxyphenyl)-acetamide (**27**), 1,6-dihydroxyphenazine (**28**), 5a,6,11a,12-tetrahydro-5a,11a-dimethyl[1,4]benzoxazino [3,2-b][1,4]benzoxazine (**29**), and keyicin (**30**).

Many of the marine bacteria used in co-cultures were derived from soil sediment. However, the literature has also shown co-cultures of sponge-derived actinomycetes [34]. Dashti et al. (2014) of the Quinn group in Griffith University co-cultured *Nocardiopsis* sp. RV163, derived from the Mediterranean sponge *Dysidea* along with *Actinokineospora* sp. EG49 from the Red Sea sponge *Spheciospongia vagabunda* to induce the biosynthesis of three compounds (Figure 6), namely, *N*-(2-hydroxyphenyl)-acetamide (**27**), 1,6-dihydroxyphenazine (**28**), and 5a,6,11a,12-tetrahydro-5a,11a-dimethyl[1,4]benzoxazino[3,2-*b*][1,4]benzoxazine (**29**), of which compound **28** was produced at a very high yield at 12% of the crude extract weight. Dashti et al. monitored the induced production of new metabolites in the co-culture by ^1^H NMR analysis of the respective culture extracts. The ^1^H NMR spectral data also indicated the suppression of the production of some of the metabolites present in the axenic cultures, which was similarly observed by Wakefield et al. in 2017 [25]. All three afforded compounds (**27** to **29**) were tested for bioactivity against *Bacillus* sp. P25, *Escherichia coli* and *Fusarium* sp. P21, and human parasites *Leishmania major* and *Trypanosoma brucei*, as well as *Nocardiopsis* sp. RV163 and *Actinokineospora* sp. EG49 cultures. Only 1,6-dihydroxyphenazine (**28**) exhibited bioactivity against *Bacillus* sp.; *Trypanosoma brucei*; and, interestingly, against *Actinokineospora* sp. EG49, which may indicate that compound **28** was biosynthesised by *Nocardiopsis* sp. RV163. These last three studies demonstrated the potential of a co-culture to induce the production of new metabolites.

The last two studies in this section are articles by the Bugni group from the University of Wisconsin. Unlike all the previous articles, these latter studies used microscale fermentation [35]. Microscale cultivation was carried out in 96-well plates, in volumes of 500 µL. Adnani et al. (2015) selected *Mycobacterium* sp. (WMMA-183) and *Rhodococcus* sp. (WMMA-185) as co-culture strains for various species from within the multiple genera of Micromonosporaceae. From the Micromonosporaceae family, 65 species were chosen for analysis of their monoculture and co-culture metabolomic profiles. While some culture samples showed antibiotic induction, some significant limitations also occurred, as this method is likely to miss antibiotics produced in the co-culture due to the initial activity observed in axenic cultures from an unrelated antibiotic. To resolve such methodological limitations, a complementary approach by LC–MS-based metabolomics was employed to analyse the types of compounds produced during the interaction and exhibition of antibiotic activity. For the 130 co-culture combinations, a total of 12 Micromonosporaceae demonstrated discernible diversity, with six producing a unique chemistry but shared similar chemical profiles when co-cultured with *Mycobacterium* sp. or *Rhodococcus* sp., while the other six produced different metabolomic profiles that were dependent on the bacterium with which it was co-cultured. Co-cultures producing similar chemical profiles were clustered, while secondary metabolites exclusively produced in co-culture were further evaluated for novelty. As an example, co-culture of *Verrucosispora* sp. and *Rhodococcus* sp. exclusively produced a total of 29 identified compounds. Of the 29 compounds, 27 were determined to be novel, and 24 could be reproduced in the scale-up. This latter study demonstrated the ability to screen and identify the generation of new metabolites from a microscale fermentation approach.

In their article, Adnani et al. (2017) described the production of keyicin (**30**), a new *bis*-nitroglycosylated anthracycline (Figure 6), by a co-culture of a *Rhodococcus* sp. and a *Micromonospora* sp. [36]. Keyicin (**30**) was produced exclusively via mixed fermentation, but physical contact between the two bacteria was not necessary. Monocultures of the two bacterial strains were incubated in distinct chambers but were connected through a tunnel separated by a diffusible membrane allowing the exchange of metabolites without any cellular transfer between chambers. Through genome sequencing, *Micromonospora* sp. was shown to be the “true” producer of keyicin in the co-culture. Keyicin (**30**) inhibited the growth of *Mycobacterium* sp. and *Rhodococcus* sp., as well as *B. subtilis* and methicillin-sensitive *Staphylococcus aureus* (MSSA) at minimum inhibitory concentrations of 8 and 2 μg/mL, respectively. In contrast to many other anthracyclines, 20 *E. coli*-based chemical genomics studies revealed that keyicin’s mechanism of action did not induce DNA damage.

In this section, it is shown that there are various types of co-culture between bacteria, and much remains to be discovered. Co-culture between bacteria has afforded interesting molecules and has made it possible to induce the production of new metabolites while increasing the production of known compounds or further enhancing their bioactivity, whether in small- to medium-sized liquid fermentation or in micro-fermentation scales. Co-culturing *Micromonospora* sp. and *Rhodococcus* sp. exclusively induced the production of the target metabolite keyicin (**30**), which was not afforded by the monocultures, although the biosynthetic gene cluster was found in *Micromonospora* sp.

### 2.3. Co-Cultures between Two Fungal Strains

In this last section, 14 pertinent articles were selected to describe the co-cultivation of two fungal species. Isolation of fungal samples from the marine environment has received increasing attention as a new source of interesting metabolites. As mentioned in the introduction, it is more difficult to differentiate a marine from a terrestrial fungus when compared to a bacterium. Unlike bacteria, marine fungi do not necessarily need sea salts in their culture medium to grow. Therefore, both obligate marine and marine-derived fungi will be included in this section.

**Figure 7:**
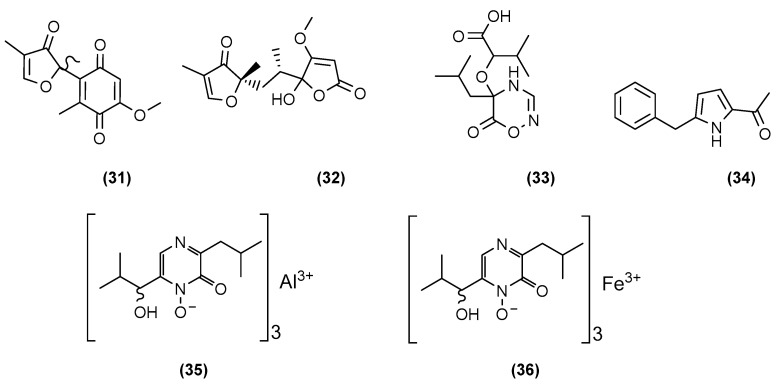
Chemical structures of sclerotiorumins A-B (**31** and **32**), sclerotiorumin C (**33**), 1-(4-benzyl-1*H*-pyrrol-3-yl)ethanone (**34**), aluminiumneohydroxyaspergillin (**35**), and ferrineohydroxyaspergillin (**36**).

Most of the literature has described fungal co-cultures between the genus *Penicillium* and/or *Aspergillus* and/or with other fungal genera. Bao et al. (2017) of the Qi group in Guangzhou, China, co-cultivated two marine gorgonian-associated fungi, *Aspergillus sclerotiorum* SCSGAF 0053 and *Penicillium citrinum* SCSGAF 0052, both isolated from *Muricella flexusa* collected from the South China Sea [37]. The development of a red pigment was only observed in the co-culture, which did not occur in either of the monocultures. Six new compounds (**31**–**36**) shown in Figure 7 were elucidated, including two furanone derivatives, sclerotiorumins A (**31**) and B (**32**); a novel oxadiazin derivative, sclerotiorumin C (**33**); a pyrrole derivative, 1-(4-benzyl-1*H*-pyrrol-3-yl)ethenone (**34**); and two complexes of neoaspergillic acid with aluminium (**35**) and iron (**36**) (Figure 7). Sclerotiorumin C (**33**) was the first naturally occurring 1,2,4-oxadiazin-6-one described. All compounds were tested for different activities—aluminiumneohydroxyaspergillin (**35**) showed significant selective cytotoxicity against human histiocytic lymphoma U937 cell line (IC_50_ = 4.2 μM) and strong toxicity towards brine shrimp (LC_50_ = 6.1 μM), and interestingly was able to increase the growth and biofilm formation of *S. aureus*.

**Figure 8:**
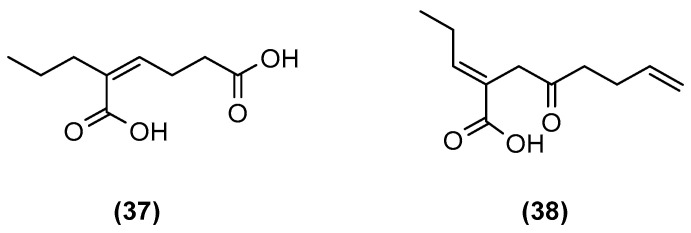
Chemical structures of (*Z*)-2-ethylhex-2-enedioic acid (**37**) and (*E*)-4-oxo-2-propylideneoct-7-enoic acid (**38**).

Kossuga et al. (2013) of the Berlinck group in São Paulo co-cultivated a total of 50 fungal strains and 5 bacterial strains in 250 growth experiments. Analysis of the extracts indicated changes in the chemical profile of eight co-cultures when compared to their corresponding monocultures. However, most of the dereplicated metabolites are already known compounds. The co-cultures produced a minority of the secondary metabolites that contained novel carbon skeletons. *Penicillium* sp. Ma(M3)V co-cultured with *Trichoderma* sp. Gc(M2)1 produced a complex extract that yielded two new polyketides (Figure 8), (*Z*)-2-ethylhex-2-enedioic acid (**37**) and (*E*)-4-oxo-2-propylideneoct-7-enoic acid (**38**) [38]. Polyketides **37** and **38** are examples of unprecedented carbon skeletons.

**Figure 9:**
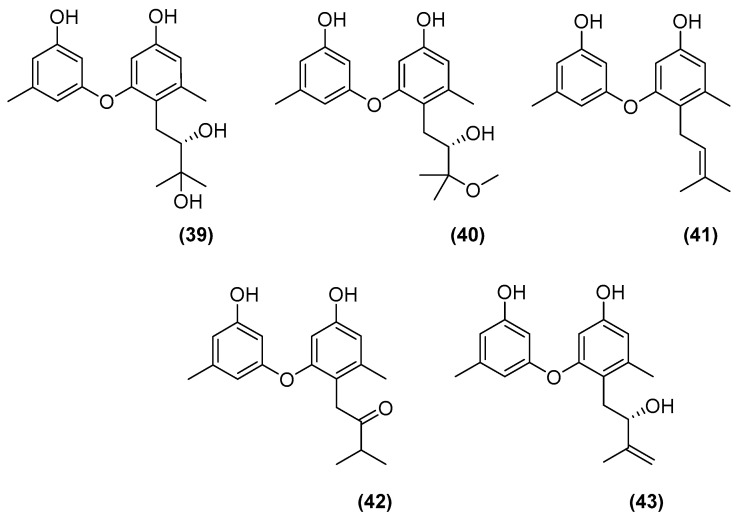
Chemical structures of diorcinols congeners B to E (**39** to **42**) and diorcinol J (**43**).

**Figure 10:**
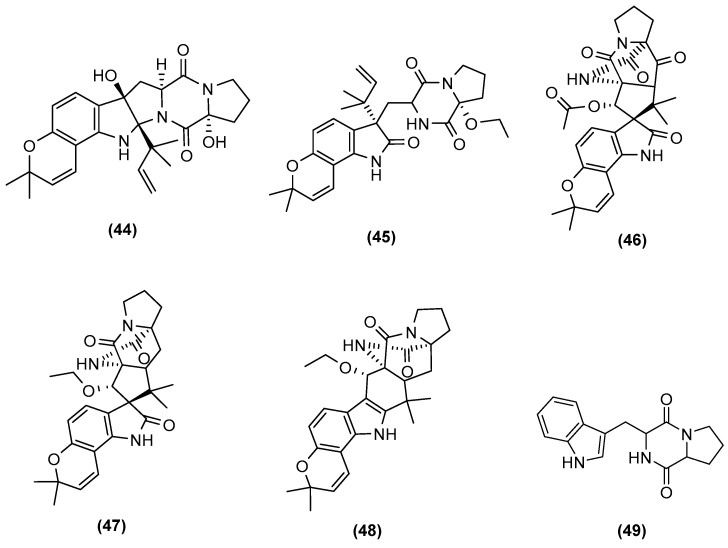
Chemical structures of 17-hydroxynotoamide D (**44**), 17-*O*-ethylnotoamide M (**45**), 10-*O*-acetylsclerotiamide (**46**), 10-*O*-ethylsclerotiamide (**47**), 10-*O*-ethylnotoamide R (**48**), and brevianamide F (**49**).

The group of Zhuravleva and Afiyatullov (2016) in Russia isolated known diorcinol congeners B to E (**39** to **42**) along with a new derivative, diorcinol J (**43**) (Figure 9), by the mixed fermentation of *Aspergillus sulphureus* KMM 4640 and *Isaria felina* KMM 4639 obtained from a muddy sand and a marine sediment, respectively [39]. Diorcinol J (**43**) was the only congener that showed cytotoxicity against Ehrlich carcinoma cells, by the membranolytic mechanism. In addition, more recently in 2018, the group of Zhuravleva and Afiyatullov isolated five new prenylated indole alkaloids (Figure 10) from the same co-culture. This included 17-hydroxynotoamide D (**44**), 17-*O*-ethylnotoamide M (**45**), 10-*O*-acetylsclerotiamide (**46**), 10-*O*-ethylsclerotiamide (**47**), and 10-*O*-ethylnotoamide R (**48**) [40]. However, 10-*O*-ethylnotoamide R (**48**) was not detected in the original extract, and thus the authors postulated that **48** might not be a natural product, but an artefact obtained during the isolation process. Zhuravleva and Afiyatullov also investigated the effect of 17-hydroxynotoamide D (**44**), 17-*O*-ethylnotoamide M (**45**), and 10-*O*-ethylnotoamide R (**48**) on the viability of human non-malignant and prostate cancer cells, as well as on the formation of colonies of human prostate cancer cells 22Rv1. 17-*O*-Ethylnotoamide M (**45**) exerted a significant effect on reducing 22Rv1 colony formation at concentrations of 10 μM by 25%. 22Rv1 cells are known to be resistant to hormone therapy, as well as to the new second-generation drugs. Therefore, drugs that are active in these cell lines may be of potential interest for further investigation in the therapy of drug-resistant human prostate cancer. Although these new molecules were produced only in the co-culture, it is worth mentioning that brevianamide F (**49**) (Figure 10), a common precursor of the afforded alkaloids, had earlier been isolated from *A. sulphureus*.

**Figure 11:**
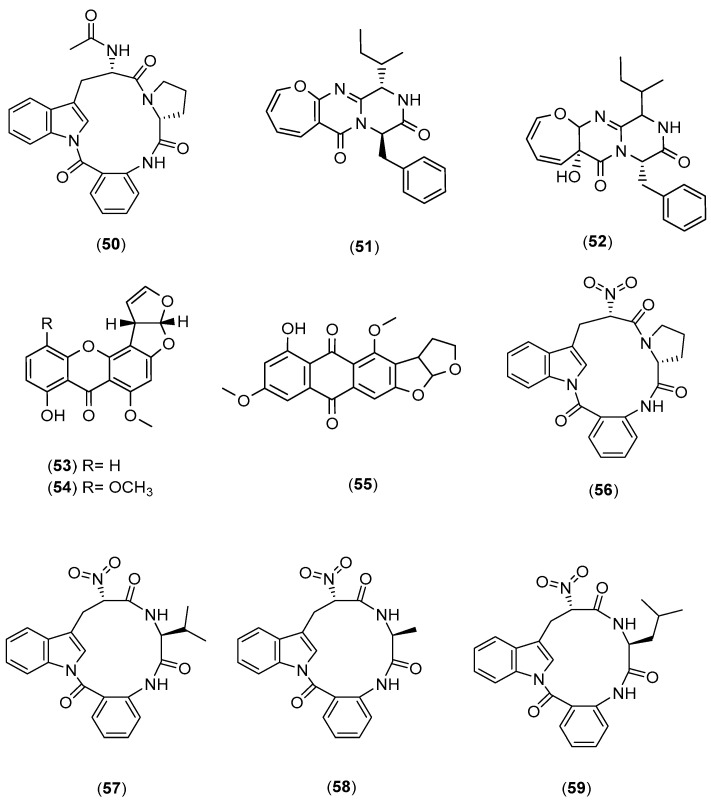
Chemical structures of psychrophilin E (**50**), protuboxepin A (**51**), oxepinamide E (**52**) sterigmatocystin (**53**), 5-methoxysterigmatocystin (**54**) aversin (**55**), and psychrophilin A to D (**56** to **59**).

Ebada et al. (2014) of the Roth group in BioMar, Düsseldorf, co-cultured two marine algal-derived fungal strains of *Aspergillus* BM-05 and BM-05ML that yielded the cyclo-tripeptide psychrophilin E (**50**), along with five known compounds (protuboxepin A (**51**); oxepinamide E (**52**); and three mycotoxins, namely, sterigmatocystin (**53**), 5-methoxysterigmatocystin (**54**), and aversin (**55**)), as shown in Figure 11 [41]. Dalsgaard et al. (2004) of the Christophersen group in Copenhagen first reported the cyclotripeptide analogues, psychrophilins A to D (**56** to **59**), which were earlier isolated from a marine-derived psychrotolerant fungal species of the genus *Penicillium* [42,43]. In contrast to previously described congeners, in psychrophilin E (**50**), the α-amino group in the tryptophan residue was acetylated into an *N*-acetyl moiety, which was oxidised in psychrophilins A to D into a nitro group. In addition, the existence of other psychrophilins in the extract could not be detected, implying that this mixed fermentation may have played a role in modifying the biosynthetic pathway by an acetylation rather than an oxidation step. All the isolated compounds from the co-culture of *Aspergillus* BM-05 and BM-05ML were assessed for their in vitro anti-proliferative activities. Psychrophilin E (**50**), sterigmatocystin (**53**), and 5-methoxysterigmatocystin (**54**) exhibited selective anti-proliferative activities towards the HCT116 (colon cancer) cell line with IC_50_ values of 28.5, 10.3, and 4.4 µM, respectively, which were more active than cisplatin as a positive control (IC_50_ = 33.4 µM).

**Figure 12:**
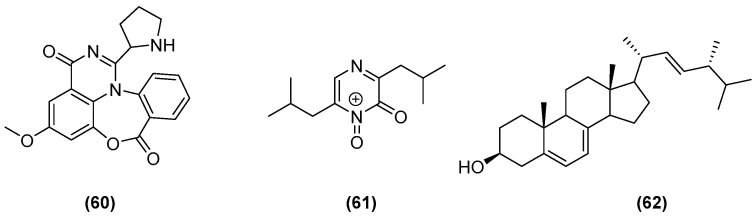
Chemical structures of aspergicin (**60**), neoaspergillic acid (**61**), and ergosterol (**62**).

The group of Zhu from Foshan University, China (2011), isolated aspergicin (**60**) with two know compounds (Figure 12), neoaspergillic acid (**61**) and ergosterol (**62**), from a co-culture of two mangrove *Aspergillus* epiphytes [44]. Both aspergicin (**60**) and neoaspergillic acid (**61**) showed significant inhibitory effect on three Gram-positive bacteria, *Staphylococcus aureus*, *Staphylococcus epidermidis*, and *Bacillus subtilis*, and three Gram-negative bacteria, *Bacillus dysenteriae*, *Bacillus proteus*, and *E. coli*, but with lower MICs for neoaspergillic acid (**61**).

**Figure 13:**
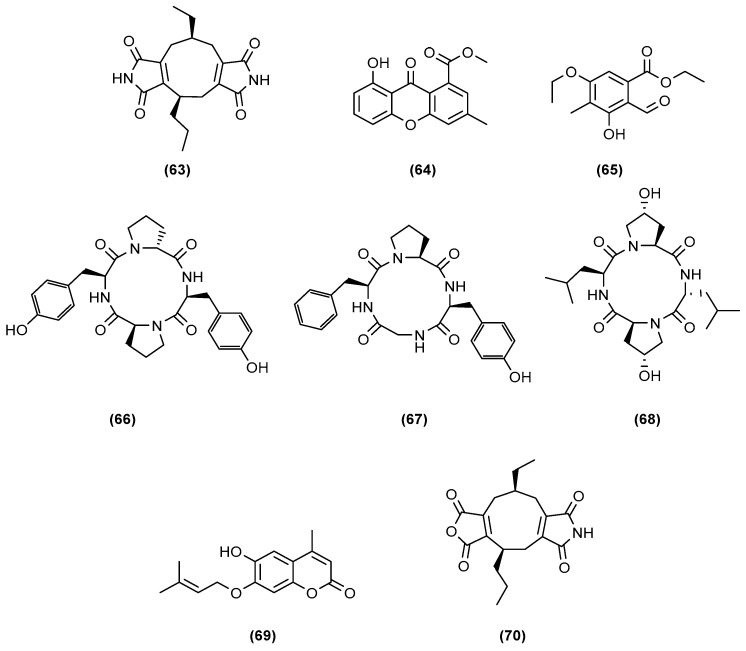
Chemical structures of (−)-byssochlamic acid bisdiimide (**63**), 8-hydroxy-3-methyl-9-oxo-9*H*-xanthene-1-carboxylic acid methyl ether (**64**), ethyl 5-ethoxy-2-formyl-3-hydroxy-4-methylbenzoate (**65**), cyclo (D-Pro-L-Tyr-L-Pro-L-Tyr) (**66**), cyclo (Gly-L-Phe-L-Pro-L-Tyr)s (**67**), cyclo-(L-leucyl-trans-4-hydroxy-L-prolyl-D-leucyl-trans-4-hydroxy-L-proline) (**68**), 7-(γ,γ-dimethylallyloxy)-6-hydroxy-4-methylcoumarin (**69**), and (−)-byssochlamic acid imide (**70**).

During the last decade, several research teams in Guangzhou, China, led by She, Lin, (2010, 2011, and 2013), and Li (2014, 2015, and 2017), have been working on secondary metabolites produced by co-cultures of *Phomopsis* sp. K38 and *Alternaria* sp. E33, and were able to isolate many new compounds. All publications from the Guangzhou teams, except for Ding et al. [45], followed the same protocol. The Guangzhou research groups collected two mangrove-derived fungi, *Phomopsis* sp. K38 and *Alternaria* sp. E33, from the South China Sea coastline. The co-culture extract displayed higher cytotoxicity against Hep-2 and HepG2 cells than that of the K38 or E33 monocultures. Amongst the first molecules isolated was a new diimide derivative (Figure 13), (−)-byssochlamic acid bisdiimide (**63**) [46]. Unfortunately, the isolated purified bisdiimide exhibited weak cytotoxicity against Hep-2 and HepG2 cells, with IC_50_ values of only 45 and 51 µg/mL, respectively. The same fungal co-culture also yielded a new xanthone derivative (Figure 13), 8-hydroxy-3-methyl-9-oxo-9*H*-xanthene-1-carboxylic acid methyl ether (**64**) [47]. The new xanthone derivative (**64**) inhibited the growth of five tested microorganisms, *Gloeasporium musae*, *Blumeria graminearum*, *Fusarium oxysporum*, *Peronophthora cichoralearum*, and *Colletotrichum glocosporioides*. The co-culture also afforded a new polysubstituted benzaldehyde, ethyl 5-ethoxy-2-formyl-3-hydroxy-4-methylbenzoate (**65**) [48]. Wang et al. (2013) bioassayed the new polysubstituted benzaldehyde compound (**65**) against four plant pathogenic fungi, namely, *Fusarium graminearum*, *Gloeosporium musae*, *Rhizoctonia solani* Kuhn, and *Phytophthora sojae* Kaufmann and Gerdemann. Antifungal activities were observed at inhibitory zone diameters of 12.06, 11.57, 10.21, and 8.50 mm, respectively.

Later studies of the same co-culture strains E33 and K38 afforded two new cyclopeptides [49]. Huang et al. (2014) evaluated the in vitro antifungal activity of two cyclic tetrapeptides, cyclo (D-Pro-L-Tyr-L-Pro-L-Tyr) (**66**) and cyclo (Gly-L-Phe-L-Pro-L-Tyr) (**67**), against *C. albicans*, *G. graminis*, *R. cerealis*, *H. sativum*, and *F. graminearum*. Both peptides **66** and **67** showed moderate-to-high antifungal activities as compared with the positive control (ketoconazole), but cyclo (Gly-L-Phe-L-Pro-L-Tyr) (**67**) was more active than cyclo (D-Pro-L-Tyr-L-Pro-L-Tyr) (**66**). Li et al. (2014) found another cyclic tetrapeptide, cyclo-(L-leucyl-trans-4-hydroxy-L-prolyl-D-leucyl-trans-4-hydroxy-L-proline) (**68**), from the same fungal co-culture [50] and tested the compound against the same crop-threatening fungi except for *C. albicans*. Compound **68** showed moderate to high antifungal activities and afforded quite a comparable bioactivity to the positive control, triadimefon, against *H. sativum*. All isolated cyclopeptides **66** to **68** are shown in Figure 13.

From the same co-culture that produced the cyclic tetrapeptides, Wang et al. (2015) isolated a new 7-(γ,γ-dimethylallyloxy)-6-hydroxy-4-methylcoumarin (**69**) (Figure 13) [51], and this was also tested against the four plant pathogenic fungi. However, unlike the peptides, the compound showed no in vitro activity against these plant pathogens over a 6.00 mm inhibition zone at 250 µM.

More recently, Ding et al. (2017) elucidated a new nonadride derivative, (−)-byssochlamic acid imide (**70**) (Figure 13), from the same mangrove fungal co-cultures [45]. Chen et al. (2004) earlier reported the antifungal, phytotoxic, enzyme inhibitory, and cytotoxic activities of nonadride analogues [52]. The new nonadride derivative from the co-culture showed moderate inhibitory activity toward *Fusarium graminearum* and *F. oxysporum* with MIC values of 50 and 60 µg/mL, respectively, as compared with the positive control carbendazim (MIC 6.25 µg/mL). By adding salt to the media, changing the incubation period and temperature, Ding et al. were able to generate a new analogue that is unrelated to cyclic tetrapeptide derivatives earlier targeted by their predecessors.

**Figure 14:**
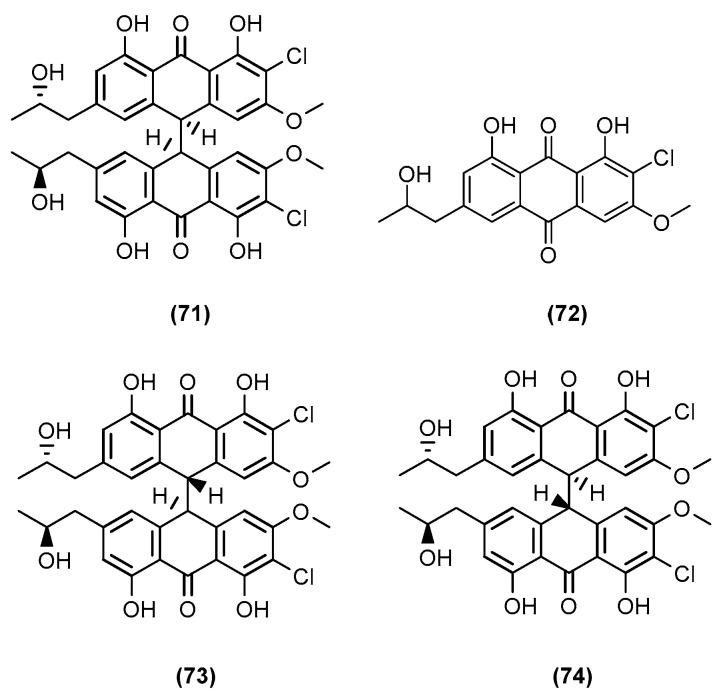
Chemical structures of allianthrone A (**71**), nalgiolaxin (**72**), allianthrone B (**73**), and C (**74**).

The last study presented in this section involved the co-culture of two fungal morphs of the marine algal-derived *Aspergillus alliaceus* [53]. The fungal co-culture consisted of identical strains but from different phases of development at their respective asexual and sclerotial morph stages. Both morphs produced distinct secondary metabolite patterns in monoculture, but their co-culture significantly changed the metabolic profile of the strain. Mandelare et al. (2018) of the Loesgen group in Oregon State isolated a new bianthrone dimer allianthrone A (**71**) from the co-cultures, while they observed elevated levels of nalgiolaxin (**72**) produced by the asexual morph. In parallel, Mandelare et al. found allianthrone A and its two diastereomers, allianthrones B (**73**) and C (**74**) (Figure 14), from the liquid fermentation culture at a ratio of 2:1:1. The two-week liquid cultures of the combined asexual and sclerotial morphs of *A. alliaceus* reliably produced the known compound nalgiolaxin at a higher yield. Allianthrone A showed weak cytotoxicity against the HCT-116 colon cancer and SK-Mel-5 melanoma cell lines. This study illustrated the first case of elicitation of new fungal chemistry by a co-culture approach of two different developmental stages of a homothallic *Aspergillus* species.

In recent publications, contrary to the previous section, it was observed that mixed fermentation between fungal species increased the discovery of new molecules, while the detection of enhanced biologically activity was less evident. Enhancing biomass yield in fungal cultures is in general easier to achieve in comparison to bacterial cultures due to their longer incubation period and life cycle. With higher biomass yield, the isolation of the metabolites is easier as well, and hence a higher rate of enhancing the production of targeted known bioactive compounds and at the same time a higher discovery rate of new compounds can be obtained but may not necessarily be active.

Nevertheless, as the utmost goal is to enhance the production yield, as well as to induce improved biological activity by stimulating the biosynthesis of new compounds, it can be discouraging that the generated new analogues may be rendered inactive. In most cases, the bioactivity seems to be at its optimum on one analogue, despite the occurrence of various new metabolites. A combination of metabolic engineering techniques needs to be considered, and this includes finding an efficient screening method to obtain the right elicitor to increase the probability of finding biologically active scaffolds. However, another limiting factor is the number of available assays in respective laboratories, and this could also be the main reason that despite the high number of new congeners being isolated, only one or two analogue(s) could be considered to exhibit potent bioactivity. The process of inducing the production of new bioactive compounds is dependent on conditions that a microorganism would need for a specific life mode to survive. There is yet much to be discovered about cryptic biosynthetic gene clusters and how they can be elicited for their expression. In this case, a full understanding of this microbial processing factory still needs to be explored.

## 3. Different Techniques of Co-Culture Used

In this last section, the techniques used by the different research teams presented in this literature review for various co-culture conditions are listed below in Table 1. The following are tabulated: the microorganisms used, their geographical origin, the culture medium(s) used, the conditions under which the cultures were made (static or under agitation, temperature, and incubation period), the experimental set-up, and the article from which the technique originated. The techniques are quite diverse, but generally the incubation time is longer for fungi than for bacteria and the temperature is often between 25 and 32 °C.

Extremely low yielding target metabolites detected in axenic cultures may not be feasibly translated for isolation work. As illustrated by the literature reviewed in this paper [24,25,26,30], these metabolites could be detected but would not be quantifiable by mass spectrometry. These same metabolites would be below the limit of detection for a UV-HPLC-coupled system. In these cases, compound yields of these target metabolites in axenic culture were not measurable prior to coculturing. The authors of the reviewed papers demonstrated enhanced compound production by comparing extracted mass ion chromatograms of the target metabolites in axenic and co-culture conditions [24,25,26,30]. As demonstrated by references listed on Table 1, successful induction of compound production afforded a range of three- to fivefold increase in yield of the target metabolite. Except for the production of lobocompactol (**23**), Cho and Kim (2012) started with a quantifiable amount of 0.25 mg/L in axenic cultures, and they achieved a 10.4-fold increase by mixed fermentation [31]. In some publications, several techniques have been tested. The techniques that have generated the most conclusive results are shown in Table 1.

## 4. Conclusions

With new omics techniques and microbiological genetic data, it has been possible to identify a variety of previously unknown metabolic pathways. Microorganisms have a very high potential to produce metabolites with a high chemical diversity. As shown in this literature review, many of the microorganisms’ biosynthetic genes remain silent or are not transcribed when they are grown under laboratory conditions. With co-culture or mixed fermentation for endogenous metabolite production, cryptic pathways can be stimulated through interaction and communication between microorganisms. Indeed, co-culture tries to mimic the environment where communications between microorganisms are abundant, dynamic, and complex.

From the various publications reviewed here, it was shown that co-culture can induce the production of new metabolites as well as increase the yields of respective target metabolites with pharmacological potential, and moreover can indirectly improve the biological activity of a crude extract. In parallel, mixed fermentation techniques may also suppress the production of metabolites that were found in axenic culture [34]. The diverse responses driven by the various co-culture methods could represent the potential counteracting response to the competition between the microorganisms. However, the afforded metabolites do not necessarily show activity against competing microorganisms, which indicates that the interactions could also be more complex than a simple question of competition.

The co-culture technique employed is essential, as it has been demonstrated that a single fermentation variable can have a very large impact on the production of specific metabolites [31]. When fungi and bacteria were co-cultured, it was essential to consider the incubation time needed for each to grow so that one does not take over the other. Physical contact may be necessary for the production of some metabolites, as for cytochalasin [24], but may not always be the case, as in the production of keyicin [36]. It is quite complicated to make any generalisations on the expected outcomes of certain co-culture techniques. However, techniques have been emerging, such as co-cultures in 96-well plates, which allowed for the testing of various combinations of media, buffers, and elicitors to be assessed. Innovative cultures also involved mixed fermentation of a single fungus from two different stages of development [53].

Furthermore, various other culture techniques could evolve to be associated with mixed fermentation methods, such as I-chip [54]. I-chip is a technique of in situ cultivation that utilises a device for the isolation of bacteria that are difficult to culture in the laboratory. This device contains hundreds of miniaturised diffusion chambers that are inoculated with a single bacterial cell. This device can then be placed in the natural environment of the organisms cultured in the diffusion chambers, be it in the sediment, the soil, or in the macro-organisms, as has recently been performed in a sponge [55]. I-chip allows microorganisms to grow in their natural environment, in addition to allocating microbial interactions, and affords access to the discovery of new organisms supplementing novel molecules [54].

The use of microorganisms from different geographical origins would also broaden the possibility to increase diversity, for example in the mixed fermentation of macro-algae endophytes simply from various parts of the globe. Microorganisms from the marine environment are still considered to be underexploited, and many discoveries are yet to be made. This technique can be used routinely in laboratories associated with techniques such as one strain many compounds (OSMAC). Natural marine products and the technique of co-culture can allow the discovery of new molecules of pharmacological interest and address the problem regarding the expression of silent genes clusters.

## Figures and Tables

**Table 1:** Widely used co-culture techniques.

Microorganisms (Experimental Aim)	Source	Media	Conditions	Experiments	Reference
*Aspergillus flavipes* *Streptomyces* sp. (enhancement of cytochalasan production)	Marine sediments of the Nanji Islands, China	5 g yeast extract, 5 g glycerol, and 1 L 75% seawater (pH 7.5)	180 rpm at 28 °C, 8 days	5 mL of microbial seed broth (*A. flavipes* and *Streptomyces* sp. in a ratio of 1:4 (*v*/*v*)) was added to the 200 mL culture medium.	[24]
*Aspergillus fumigatus* MR2012 *Streptomyces leeuwenhoekii* (inducing the generation of new compounds and increasing the yield of existing metabolites)	Red sea sediment in Hurghada, Egypt. Hyper-arid soil of Laguna de Chaxa, Chile	ISP2 medium (4.0 g yeast extract, 10.0 g malt extract, 4.0 g dextrose in artificial sea water; pH 7.2)	180 rpm at 30 °C, 8 days	200 mL of primary seed culture of each of fungal and bacterial isolates was used to inoculate 4 L of ISP2. Inoculation of the primary fungal culture was started 2 days before bacterial inoculation.	[25]
*Bacillus* sp., *B. pumilus*, *B. licheniformis*, *Serratia marcescens* with *Candida albicans*, *Yarrowia lipolytica*, *Pseudomonas aeruginosa* (induction and enhancement of the production of certain bioactive metabolites)	Surfaces of the green mussel, *Perna viridis* and the coral, *Symphyllia* sp. from the nearshore regions of Kovalam and Mandapam, Tamil Nadu, India.	LB medium (10 g peptone, 10 g NaCl, 5 g yeast extract, for 1 L; pH = 7)	30 °C, 24 h	10 µL (1 × 10^8^ cells/mL) of 12-h-old culture of inducer fungi or bacteria was added to the flasks containing 12-h-old culture of marine isolates.	[26]
*Streptomyces* sp. (reported biological activity was enhanced by 70% with the crude extract of the co-culture, but there is no record whether new compounds were generated or merely enhanced concentration of biologically active components)	Soil of mangrove forest Sundarbans, Bangladesh	Yeast extract glucose broth media (yeast extract 2.5 g/L, glucose 5 g/L)	220 rpm at 31 °C, 7 days	20 mL inocula (2 days of fermentation) of both fungi were mixed in a 500 mL conical flask containing 200 mL sterilised yeast-extract glucose broth media (co-culture).	[28]
*Streptomyces* sp. *Bacillus mycoides* (enhancement of the production of a target metabolite)	Marine sediments of the Nanji Islands, China	MM medium (5 g yeast extracts, 5 g glycerol in 1 L 75% sea water; pH 8.0)	Static incubation, 14 days	*Streptomyces* sp. was first cultivated in 500 mL Erlenmeyer flasks containing 200 mL of MM medium for 7 days, then 1% (*v*/*v*) of *B. mycoides* suspension (OD_590_ 0.5) was added.	[30]
*Streptomyces cinnabarinus* PK209 *Alteromonas* sp. KNS-16 (enhancement of the production of a target metabolite)	Sediments and seaweed rhizosphere, depth of 10 m along coast of Korea	TBFeC medium (3 g tryptone, 5 g casitone, 4 g of glucose, 0.04 g Fe_2_(SO_4_)_3_ 4H_2_O, 0.1 g KBr, and 1 L of sea water; pH 7.8)	215 rpm at 25°, 288 h	1 mL (10^5^ cells) of 16-h-old KNS-16 culture in NB medium was inoculated into 1 L of 96-h-old strain PK209 in TBFeC medium in Fernbach flasks.	[31]
*Streptomyces* sp. *Bacillus* sp. (to induce production of dentigerumycin E)	Mud sample from intertidial mudflat in Wando, Republic of Korea	YEME liquid medium (4 g yeast extract, 10 g malt extract, 4 g glucose in 1 L artificial seawater)	200 rpm at 30°, 8 days	Equal volumes of 4-day cultures of both fungi were mixed (10 mL to 10 mL) and inoculated into a 500 mL baffled Erlenmeyer flask containing 200 mL of YEME liquid medium.	[32]
*Janthinobacterium* spp. ZZ145 and ZZ148 (to induce the generation of the new janthinopolyenemycin congeners)	Marine soil from coastal area of Sindh, Karachi, Pakistan	Rice medium (rice 40 g, sea salt 35 g, tap water 60 mL)	Under stationary state at 28°, 25 days	3.5 mL of ZZ145 in EY liquid medium and 3.5 mL of ZZ148 in B liquid medium were inoculated into rice medium in 500 mL Erlenmeyer flasks.	[33]
*Actinokineospora* sp. EG49 *Nocardiopsis* sp. RV163 (to induce the generation of new metabolites.)	*Spheciospongia vagabunda* (Red Sea sponge) *Dysidea avara* (Mediterranean sponge)	ISP2 medium (4.0 g yeast extract, 10.0 g malt extract, 4.0 g dextrose in artificial sea water; pH 7.2)	150 rpm at 30°, 7 days	10 mL of 5-day-old culture of *Nocardiospsis* was inoculated into 2 L Erlenmeyer flasks, each containing 1 L of ISP2m inoculated with 10 mL of 5-day old culture of *Actinokineospora*.	[34]
*Mycobacterium* sp. *Rhodococcus* sp. (screen and identify the generation of new metabolites)	Sponge or ascidian specimens in the Florida Keys, USA	ASW-A media (20 g soluble starch, 10 g glucose, 5 g peptone, 5 g yeast extract, 5 g CaCO_3_ per litre of artificial seawater)	300 rpm at 30°, 14 days	In detoxified polypropylene square 96-deepwell microplates, 500 μL ASW-A was added to each well. Wells were inoculated with 15 μL of *Micromonosporaceae* and 5 μL of mycolic acid-containing bacteria.	[35]
*Rhodococcus* sp. *Micromonospora* sp. (to induce the generation of a new metabolite)	Marine sponge *Chondrilla nucula* and ascidian *Ecteinascidia turbinata*	ASW-D media (2 g yeast extract, 5 g malt extract, 2 g dextrose per litre of artificial seawater)	14 days	Same techniques used in [28].	[36]
*Aspergillus sclerotiorum* *Penicillium citrinum* (to induce the generation of new analogues)	Gorgonian *Muricella flexuosa* collected from the South China Sea, Sanya	Glucose 1.0%, MgSO_4_ 0.1%, KH_2_PO_4_ 0.1%, peptone 0.1%, sea salt 3.0% and pH 6.5–7.0	Static incubation at 28°, 30 days	1 mL, about 10^8^ CFU/mL of *P. citrinum*, and 1 mL, about 10^4^ CFU/mL of *A. sclerotiorum* were inoculated into 1 L flasks containing 300 mL of liquid medium.	[37]
*Penicillium* sp. *Trichoderma* sp. (to induce the generation of new analogues)	*Mycale angulosa* *Geodia corticostylifera* (marine sponges)	Malt medium (20 g malt extract, ASW 1 L; pH 8.0)	100 rpm at 25°, 12 days	8 plugs of mycelia of each fungus, grown in Petri dishes, were inoculated in 250 mL of 2% malt medium.	[38]
*Aspergillus sulphureus* *Isaria eline* (to induce the generation of new analogues)	Muddy sand of eastern Sakhalin shelf and sediments of South China Sea	20 g rice, 20 mg yeast extract, 10 mg KH_2_PO_4_, 10 mg KNaC_4_H_4_O_6_ 4H_2_O and 40 mL natural seawater	25°, 14 days	*A. sulphureus* was cultivated for 7 days, then inoculated with *I. eline*, and co-cultivated.	[39]
		20 g of rice, 20 mg yeast extract, 10 mg KH_2_PO_4_, and 40 mL of natural sea water	14 days	They were grown separately for 7 days and then *I. eline* mycelium was inoculated into 20 flasks with *A. sulphureus* culture.	[40]
*Aspergillus* sp. (to induce the generation of new analogues)	*Sargassum* collected off Helgoland, North Sea Germany	Peptone from soya 4 g, maize starch 10 g, MgSO_4_ 3.6 g, NaCl 20 g, yeast extract 2 g, CaCO_3_ 1.8 g, per 1 L demineralised water	Static incubation at 28°, 28 days	Agar plugs from plated cultures were co-cultivated in 1 L Erlenmeyer flasks (500 mL/flasks).	[41]
*Aspergillus* sp. (to induce the generation of new analogues)	Rotten fruit of a mangrove *Avicennia marina* in Zhanjiang, China	GYP medium (glucose 10 g/L, yeast extract 1 g/L, peptone 2 g/L, crude sea salt 3.5 g/L; pH 7.0)	Room temperature, 30 days	Inoculated with the mycelium of the isolate FSY-01, then inoculated with that of FSW-02 immediately.	[44]
*Phomopsis* sp. K38 *Alternaria* sp. E33 (to induce the generation of new analogues)	Mangrove in Leizhou Peninsula, Guangdong Province, China	Glucose 10 g/L, peptone 2 g/L, yeast extract 1 g/L, NaCl 30 g/L	30°, 25 days	Plugs of agar supporting mycelial growth were cut and transferred to a 250 mL Erlenmeyer flask containing 100 mL of the liquid medium. After 5–7 days, the mycelium was transferred to 500 mL Erlenmeyer flasks containing 200 mL of culture liquid.	[46,47,48,49,50,51]
		GYT medium (1% glucose, 0.1% yeast extract, 0.2% peptone, 0.2% crude sea salt)	Static incubation at 28°, 30 days	A small scrap of an agar slice with mycelium was added into a 500 mL Erlenmeyer flask containing 250 mL of GYT medium.	[45]
*Aspergillus alliaceus* (elicitation of new fungal chemistry)	Marine alga	Malt pH 6 buffered (malt extract 20 g/L, glucose 10 g/L, yeast extract 2 g/L, (NH_4_)_2_HPO_4_ 0.5 g/L)	110 rpm at 28°, 30 days	Both developmental stages of *A. alliaceus* were grown on separate agar plates and used to inoculate each 50 mL of malt liquid media. After 2 weeks, the two cultures were combined into 1 L of malt-based buffered media.	[53]

EY liquid medium: yeast 1.0 g, tryptone 5.0 g, FeCl_3_·6H_2_O 0.17 g, KH_2_PO_4_ 0.12 g, sea salt 35 g, water 1 L. B liquid medium: soluble starch 20 g, KNO_3_ 1 g, K_2_HPO_4_ 0.5 g, MgSO_4_·7H_2_O 0.5 g, NaCl 0.5 g, FeSO_4_ 0.01 g, water 1 L. Legend: LB medium = Luria–Bertani medium; YEME medium = yeast extract–malt extract medium; ISP2 medium = International *Streptomyces* Project-2 medium; ASW = artificial seawater medium.
